# Forebrain-Midbrain Circuits and Peptides Involved in Hyperalgesia After Chronic Alcohol Exposure

**DOI:** 10.35946/arcr.v41.1.13

**Published:** 2021-10-14

**Authors:** Nicholas W. Gilpin, Waylin Yu, Thomas L. Kash

**Affiliations:** 1 Department of Physiology, Louisiana State University Health Sciences Center, New Orleans, Louisiana; 2 Department of Neuroscience, Louisiana State University Health Sciences Center, New Orleans, Louisiana; 3 Alcohol and Drug Abuse Center of Excellence, Louisiana State University Health Sciences Center, New Orleans, Louisiana; 4 Biomedical Laboratory Research and Development and Clinical Science Research and Development Intramural Program, Southeast Louisiana Veterans Health Care System, New Orleans, Louisiana; 5 Department of Pharmacology, Bowles Center for Alcohol Studies, University of North Carolina at Chapel Hill School of Medicine, Chapel Hill, North Carolina

**Keywords:** alcohol, pain, withdrawal, dependence, hyperalgesia, allodynia

## Abstract

People living with pain report drinking alcohol to relieve pain. Acute alcohol use reduces pain, and chronic alcohol use facilitates the emergence or exaggeration of pain. Recently, funding agencies and neuroscientists involved in basic research have turned their attention to understanding the neurobiological mechanisms that underlie pain-alcohol interactions, with a focus on circuit and molecular mediators of alcohol-induced changes in pain-related behavior. This review briefly discusses some examples of work being done in this area, with a focus on reciprocal projections between the midbrain and extended amygdala, as well as some neurochemical mediators of pain-related phenotypes after alcohol exposure. Finally, as more work accumulates on this topic, the authors highlight the need for the neuroscience field to carefully consider sex and age in the design and analysis of pain-alcohol interaction experiments.

Chronic pain increases the risk for development of alcohol use disorder (AUD). Given that acute alcohol consumption can reduce pain, humans sometimes drink alcohol for relief of pain. Chronic alcohol consumption, however, can increase pain sensitivity during withdrawal and facilitate pain sensitization related to comorbid pain conditions. 1 Ascending and descending nociceptive circuitry and higher order pain processing centers exhibit a high degree of overlap with the brain circuits that mediate behaviors associated with alcohol reinforcement and with AUD, alcohol dependence and withdrawal.^1^ Recently, the National Institute on Alcohol Abuse and Alcoholism has prioritized research that aims to uncover the neurobiological mechanisms that underlie pain-associated increases in alcohol drinking (i.e., self-medication) and the emergence or exaggeration of pain phenotypes by chronic alcohol exposure. Layered into this research topic are questions of sex differences and species differences, age at time of alcohol exposure and testing, and potential effects of dose, duration, route, and pattern of alcohol exposure. In response to this programmatic shift, neuroscientists involved in basic research have increased their investigation of the neurobiological mechanisms underlying these phenomena, including circuit interrogation and testing the role of neuropeptides, which are the two focal points of this review. Discussed below are the roles of two circuits between the limbic forebrain and the midbrain, more specifically, projections descending from the central amygdala (CeA) to the periaqueductal gray (PAG) and projections ascending from the PAG to the bed nucleus of the stria terminalis (BNST), as well as peptidergic transmission within those regions and circuits, in mediating alcohol-pain interactions. Although each of these brain regions can be subdivided, the literature in this area is in its nascent stages and in many cases the contributions of subregions have not been examined; therefore, this review’s language regarding brain regions reflects the resolution provided within the cited studies.

## SUPRASPINAL CIRCUITS IN ALCOHOL WITHDRAWAL HYPERALGESIA

In the brain, the molecular and cellular mechanisms involved in pain processing are complex and diverse. The ventrolateral PAG (vlPAG) receives information from ascending pain pathways and is a major source of descending outputs responsible for inhibitory control of pain.^2^,^3^ In adult male and female Sprague-Dawley rats, the analgesic and antihyperalgesic effects of opioid receptor agonists are in part attributed to their action in the PAG.^4^ More specifically, opioid drugs can reduce pain in adult male Sprague-Dawley rats by facilitating the activity of descending vlPAG outputs to the rostral ventrolateral medulla (RVM),^5^,^6^ and evidence suggests that endogenous opioids may modulate pain similarly in rodents.^7^ In adolescent male Swiss Webster mice, mu opioid receptor activation also disinhibits tyrosine hydroxylase (TH)-positive (i.e., dopaminergic [DAergic]) neurons in the PAG via local gamma-aminobutyric acid-ergic (GABAergic) inputs.^8^ Therefore, the PAG receives input from ascending pain modulation pathways, has reciprocal connections with the limbic forebrain, and mediates the analgesic effects of opioid drugs via multiple mechanisms and circuits. Furthermore, chronic alcohol exposure produces plasticity in the CeA^9^ and BNST,^10^ both of which are inputs to the PAG,^11^,^12^ suggesting that the same circuitry responsible for pain modulation may be sensitive to prolonged alcohol use.

A majority of prior work examining the neurobiology of alcohol has focused on the investigation of individual brain regions, but progressively more attention is being paid to the acute and chronic effects of alcohol on brain circuits involved in nociception. The ultimate goal of circuit-level analysis of alcohol-related hyperalgesia is to facilitate the identification of potential treatment targets in humans with AUD living with pain. This may be achieved by establishing the molecular signature of cells that modulate pain and nociception via projections to other brain regions. For example, if specific receptor subtypes are preferentially enriched on a subset of projection neurons, then pharmacological modulation of those receptors may present a unique opportunity to modulate that circuit for reducing pain-related outcomes with minimal off-target effects.

## VLPAG/DR TO BNST:AN ASCENDING ANTINOCICEPTIVE CIRCUIT

Dopamine (DA) neurons in the vlPAG/dorsal raphe (DR) were first characterized in a series of neuroanatomical studies, where they were reported to be a dorso-caudal extension of the ventral tegmental area (VTA).^13^,^14^ Dopamine neurons in the vlPAG/DR are disinhibited by mu opioid receptor agonists and have roles in mediating pain and arousal in rats and mice of various strains and ages.^8^,^14^–^22^ Notably, the vlPAG/DR and VTA project to the extended amygdala, where co-release of DA and glutamate activates neurons in the BNST and the CeA of rats and mice.^8^,^23^–^24^ Therefore, it is reasonable to postulate that DA signaling in these extended-amygdala structures alters some aspects of the pain experience.

In support of this notion, bath application of alcohol promotes firing of vlPAG/DR DA neurons in brain slices taken from adolescent male Swiss Webster mice, potentially via modulation of glutamatergic transmission.^25^ Furthermore, systemic administration via intraperitoneal injection of alcohol or morphine can increase extracellular DA levels in the BNST of presumably adult (230–250 g) male Sprague-Dawley rats,^26^ and morphine increases phosphorylation of extracellular signal–regulated kinase (ERK) via dopamine D1 receptors in adult male and female C57 mice,^27^ suggesting that drugs of misuse may modulate pain via activation of DA inputs to the BNST. Given the role of the BNST in regulating emotional and motivational behaviors, vlPAG/DR DAergic projections to the BNST may mediate or mitigate the affective aspects of chronic pain. Interestingly, both chemogenetic activation of vlPAG/DR DAergic neurons and optogenetic activation of vlPAG/DR TH-positive outputs to the BNST are antinociceptive in adolescent^8^ and adult^20^,^28^ male mice. This antinociceptive effect manifests as a reduction in basal pain sensitivity and attenuation of hypersensitivity after persistent intraplantar inflammation, demonstrating a possible role for DAergic projections from the vlPAG/DR to the BNST in pain-related outcomes.

## THE VLPAG/DR-BNSTCIRCUIT IN SEX-SPECIFIC MECHANISMS OF PAIN

In humans, pain drives greater functional connectivity between the PAG and limbic structures in men relative to women,^29^ but the mechanisms behind these differences are unclear. Only a few molecular drivers of sex differences have been identified for pain,^30^ with some evidence supporting a role for DA signaling in the vlPAG/DR and the BNST. In the midbrain, the vlPAG-RVM circuit is critical for mediating morphine antinociception and tolerance to this effect in adult male Sprague-Dawley rats;^4^ the same effects are facilitated, at least in part, by morphine-microglia interactions in adult female Sprague-Dawley rats.^31^ Chronic inflammatory pain increases presynaptic GABA release but decreases high-affinity tonic gamma-aminobutyric acid type A (GABA_A_) receptor-mediated currents exclusively in vlPAG neurons of adolescent female Sprague-Dawley rats, an effect that may be associated with sex-specific morphine-induced antinociception, which was measured in adult Sprague-Dawley rats in the same study.^32^ These data suggest that opioids and chronic inflammation alter GABAergic signaling in the vlPAG and influence pain sensitivity differently for males and females. Considering that the same vlPAG GABAergic neurons that regulate the activity of RVM-projecting cells also may govern the activity of vlPAG DAergic neurons in adolescent and adult mice,^8^,^22^ it is possible that BNST-projecting vlPAG/DR DA-positive cells contribute to sex differences in pain processing. Previous work from the Kash lab and others have shown that DAergic cells in the vlPAG/DR robustly project to the BNST and that their activation reduces pain-related behaviors in adolescent and adult male mice,^8^,^20^ but these evaluations were performed only in male mice and did not examine circuit activity. Newly published data from the Kash lab indicate that optogenetic activation of DAergic inputs from the vlPAG/DR to the BNST alters nociception in adult male but not female C57 mice,^28^ and that this activation is associated with subtle changes in dopamine receptor function assessed in brain slices. In contrast, local antagonism of DA D1 receptor in the BNST increases pain-like behavior only in adult female rats.^33^ These data suggest not only sex differences, but also species differences, in the role of DAergic inputs from the vlPAG/DR to the BNST in mediating pain-related behavior.

## DA REGULATION OF CRF FUNCTION IN THE BNST, SEX, AND REGULATION OF PAIN

The BNST is a center of integration for value representation, motivated behaviors, threat response, and drug use.^34^ Although the potential role of the BNST in pain is not well characterized, it has been suggested that corticotropin-releasing factor (CRF) signaling in the BNST has a role in the sensory and affective-motivational components of pain,^35^–^41^ which parallels data showing that CRF signaling in the CeA of adult male Sprague-Dawley rats facilitates pain-like responses via actions at CRF type-1 receptor (CRFR1).^42^ Evidence for DA-CRF interactions comes from findings that DA enhancement of glutamatergic synaptic transmission in the BNST is regulated by CRFR1 activity in adolescent male C57 mice.^43^ Although it has been assumed that this DA comes from the VTA, the vlPAG/DR remains an intriguing possibility as the source of DA in the BNST. Because DA neurons in the vlPAG/DR co-express vasoactive intestinal peptide (VIP) in mice^44^ and VIP neurons in the vlPAG/DR terminate onto CRF neurons in the BNST, vlPAG/DR DA neurons may directly influence CRF signaling by innervating CRF neurons in the BNST. A more direct indication by Meloni et al. (2006)^45^ shows that in adult male Sprague-Dawley rats, the majority of DA neurons innervating CRF neurons in the BNST originate in the vlPAG/DR. Therefore, vlPAG/DR DA neurons may interact with CRF signaling via direct cellular transmission onto CRF neurons in the BNST. Finally, BNST anatomy, CRF distribution,^46^ DA modulation of pain^33^ and behavioral effects^47^ differ in male and female rats and mice. Therefore, it is possible that DA cells in the vlPAG/DR and CRF cells in the BNST work together to contribute to sex differences in pain. The Kash lab has begun to explore this possibility using in vivo imaging.^48^ Briefly, the authors found that CRF neurons in the BNST are dynamically engaged during nociceptive processing; however, there is reduced activation of BNST CRF neurons during noxious heat exposure in adult female mice compared to males. It will be critically important to determine if the dynamics of CRF neurons in the BNST are altered following alcohol exposure. Although there has been more focus on the role of dopaminergic innervation of the BNST in pain-related outcomes, one study reported that activation of serotonergic projections from the DR to the CeA reduces negative affective behavior in adult male mice with chronic inflammatory or neuropathic pain.^49^ This finding is especially intriguing given that selective serotonin reuptake inhibitors have been used to treat both AUD and pain disorders.

## CEA TO VLPAG PROJECTIONS IN ALCOHOL WITHDRAWAL HYPERALGESIA

The vlPAG is densely innervated by descending inputs from the CeA.^50^,^51^ In the context of chronic pain models, the CeA and its projections to the vlPAG have been tested for their role in pain-related behaviors in adult and adolescent rats and mice.^52^–^56^ Early studies showed that electrical stimulation of the CeA in rodents produces analgesia, and that this effect is blocked by lidocaine-induced inactivation of PAG or by opioid receptor blockade in PAG, suggesting that cells projecting from the CeA to the PAG modulate the nocifensive response in male Wistar rats weighing 140–160 grams.^57^ Data from the Gilpin lab show that inactivation of the CeA as a whole (using tetrodotoxin),^58^ or inactivation of cells projecting from the CeA to the vlPAG (using optogenetics),^11^ is pronociceptive in adult male Wistar rats. Work from other groups shows that ERK activation in the amygdala (manipulations aimed at the CeA) is necessary and sufficient to induce lasting mechanical hypersensitivity in male Swiss Webster mice weighing 40–45 grams.^54^ In recent years, it has become increasingly clear that the role of the CeA in mediating pain-like responses is affected by the CeA subregion (medial versus lateral division)^55^ and CeA cell type (according to their morphology, electrophysiology and molecular signature)^52^ being examined, as well as laterality of pain and amygdala,^53^ and possibly also by chronic pain state, species, sex, and age of experimental subjects.

Based on this prior work, the Gilpin lab investigated the relationship between chronic exposure to high-dose alcohol, CeA-vlPAG circuit activity, and pain-related outcomes in rats. Adult male Wistar rats rendered alcohol-dependent via long-term alcohol vapor exposure (i.e., exposure models that produce physiological and behavioral signs of withdrawal upon termination of alcohol exposure) exhibit thermal hyperalgesia during withdrawal, which is not observed in rats that are nondependent alcohol drinkers or alcohol-naïve controls.^59^ This effect is reversed by acute bolus systemic alcohol injections and by oral alcohol self-administration prior to nociception testing.^59^ In subsequent work using optogenetic stimulation of CeA terminals in the vlPAG, alcohol-dependent adult male Wistar rats exhibited weaker connectivity between the CeA and the vlPAG during alcohol withdrawal, as evidenced by lower amplitude of inhibitory postsynaptic currents.^11^ The authors also showed that pro-nociceptive manipulations in the CeA of rats can be reversed by mu opioid receptor blockade in the vlPAG (confirming the result found by Oliveira and Prado in 2001,^57^ mentioned above), that optogenetic activation of CeA neurons projecting to the vlPAG attenuates hyperalgesia associated with alcohol withdrawal in alcohol-dependent rats, and that inhibition of these neurons produces thermal hyperalgesia in otherwise experimentally naïve adult male Wistar rats.^11^

## AMYGDALAR MC4R SIGNALING IN ALCOHOL WITHDRAWAL HYPERALGESIA

Chronic alcohol exposure and withdrawal alters melanocortin 4 receptor (MC4R) expression in the CeA of adult male Wistar and Sprague-Dawley rats,^11^,^60^ and site-specific antagonism of MC4Rs in the CeA reverses alcohol withdrawal hyperalgesia in adult male Wistar rats.^11^ Antagonism of MC4Rs in the amygdala facilitates the antinociceptive effects of morphine and prevents the development of tolerance to the analgesic effects of morphine as well as the emergence of paradoxical hyperalgesia during morphine withdrawal in adult male Sprague-Dawley and Wistar rats.^61^,^62^ Furthermore, MC4R antagonism reduces neuropathic pain in adult male Wistar rats.^63^,^64^

MC4Rs are expressed at most levels of the ascending and descending pain circuitry and induce plasticity by altering trafficking of alpha-amino-3-hydroxy-5-methyl-4-isoxazolepropionic acid (AMPA) receptors to the membrane.^65^ Therefore, it would be of interest to know whether MC4R expression is enriched specifically in cells linking brain regions important for pain processing, that is, in pain circuits (e.g., on the postsynaptic membranes of vlPAG-projecting cells in the CeA). It is unclear whether MC4Rs are expressed or enriched on the postsynaptic membrane of vlPAG-projecting cells in the CeA. Ongoing work is examining whether this is the case and aims to test whether modulation of MC4R activity on vlPAG-projecting CeA cells attenuates alcohol withdrawal hyperalgesia and other pain states. Collectively, there is growing literature showing that MC4R antagonism in the CeA—and other sites in the central nervous system—reduces pain-related behavior in multiple pain states. This is exciting when one considers that intranasal delivery of an MC4R antagonist blocks alcohol withdrawal hyperalgesia in adult male Wistar rats^59^ and reduces anxiety-like behavior in male Sprague-Dawley rats weighing 150–160 grams,^66^ suggesting that intranasal delivery of MC4R antagonists to treat pain conditions may hold promise for translation to the clinic.

## AMYGDALAR CRF SIGNALING IN ALCOHOL WITHDRAWAL HYPERALGESIA

Many years of research have been devoted to understanding the behavioral effects of CRFCRFR1 signaling in the CeA. Much of this work has focused on addiction-related behaviors, anxiety-like behavior, stress reactivity, fear-related behavior, and nociception in rats and mice.^9^,^56^,^67^ CRF and CRFR1 messenger RNA (mRNA) and protein levels are highly expressed in the CeA of adult male Wistar rats,^68^ CRF increases inhibitory transmission in the CeA, and this effect is altered by alcohol dependence in adolescent male Sprague-Dawley rats.^69^ In the same study, chronic in vivo systemic CRFR1 antagonism during alcohol withdrawal prevented the emergence of dependence-like phenotypes during subsequent withdrawals in adult male Wistar rats.^69^ Antagonism of CRFR1 in the CeA acutely reduces anxiety-like behavior in adult male Wistar rats,^70^ reduces avoidant behaviors in adult male Wistar rats with high stress reactivity,^71^ reduces escalation of alcohol drinking in alcohol-dependent and stressed adult male Wistar rats,^68^,^71^ and attenuates hyperalgesia induced by nicotine dependence and predator odor stress in adult male Wistar rats.^58^,^72^

Recent work from the Gilpin lab shows that chronic alcohol exposure during adolescence leads to hyperalgesia and reductions in synaptic drive onto vlPAG-projecting CeA cells in rats, effects that last many weeks after termination of alcohol exposure in male but not female Wistar rats.^73^ This latter finding is in agreement with prior work from the authors showing weaker CeA-vlPAG connectivity in alcohol-dependent adult male Wistar rats that are hyperalgesic during acute withdrawal.^11^ It remains to be determined whether the effects of CRFR1 in the CeA on alcohol withdrawal hyperalgesia can be attributed to their expression on specific subsets of CeA projection cells. Ongoing work by the authors seeks to build on these initial circuit-level findings by determining (1) how the CeA-vlPAG circuit is modulated by the activity of specific peptide systems during alcohol withdrawal, and (2) the role of these cell type-specific circuits in mediating alcohol withdrawal hyperalgesia.

## BIOLOGICAL FACTORS IN ALCOHOL WITHDRAWAL HYPERALGESIA

Men and women experience, process, and report pain differently.^74^,^75^ Similarly, rodents exhibit sex differences in baseline nociception and responses to the antinociceptive effects of analgesic drugs.^76^ Under healthy and chronic pain conditions, humans and non-human animals exhibit diffuse noxious inhibitory control (DNIC) of pain (also called inhibition of pain by pain) that is modulated by sex.^77^,^78^ In rats, DNIC is less efficient in females compared with males, and the brain networks engaged during DNIC differ in males and females.^77^ The above discussion of sex differences in pain modulation by vlPAG/DR circuit projections to the BNST is a good example of why it is critical for the nascent alcohol-pain field to include both sexes in all studies.

It is largely unknown how the age of onset for chronic pain affects (1) pain-induced alterations in the central nervous system, (2) neurobiological mediators of the effects of pain on behavior, and (3) the modality, intensity, and duration of chronic pain–related behavior. For example, in the CeA, some studies of chronic inflammatory pain have been performed in adult mice,^52^ and others have been performed in adolescent mice.^55^ In those studies, CeA cells were sorted and classified according to firing pattern (e.g., regular spiking, fast spiking, late firing, bursting), and the resulting cell population breakdown differed greatly between the two studies. Thus, even when measuring similar physiological outcomes in vitro in the CeA of mice treated with the same in vivo manipulation (i.e., CFA to induce chronic inflammatory pain), results may vary. There are several possible explanations for these discrepant results, but one potential major contributor to these results is the age of rodents at the time of CFA treatment and sacrifice. In the context of pain-alcohol relationships, age may be especially important because (1) alcohol effects on the central nervous system differ according to age of exposure, and (2) human data show that the relationship between pain severity and alcohol use begins early in life, and that childhood trauma is associated with increased risk of chronic pain in adulthood.^79^,^80^ As mentioned above, chronic alcohol exposure during adolescence produces mechanical hypersensitivity and thermal hyperalgesia that last for many weeks following termination of alcohol exposure, but the underlying neurobiological mediators of these effects are unknown. As more research is devoted to testing the neurobiological mechanisms underlying pain-alcohol interactions, it will be important for the field to pay close attention to sex and age differences.

## CONCLUSIONS

Neuroscience has recently turned its attention to understanding the neurobiological mechanisms that underlie pain-alcohol interactions. This new research has primarily focused on the emergence of pain-like states after chronic alcohol exposure using animal models, but it also should focus on alcohol use and alcohol effects in rodents with chronic inflammatory or neuropathic pain. To this point, work has focused largely on circuit and molecular mediators of alcohol-related hyperalgesia. Above, this review discusses examples of recent work in this area, with a focus on reciprocal projections between midbrain and the limbic forebrain (i.e., extended amygdala) as well as some neurochemical mediators (dopamine, melanocortins, and CRF) of pain-related phenotypes after alcohol exposure. This list undoubtedly will grow as more labs begin to work in this area, and it will be important going forward for the field to be mindful of sex and age (as well as species) in study design and data analysis of pain-alcohol interactions.
